# Evidence based care of type 1 diabetes in the asia pacific region

**DOI:** 10.1186/1687-9856-2013-S1-O11

**Published:** 2013-10-03

**Authors:** Maria Craig

**Affiliations:** 1 Paediatric Endocrinologist, Sydney, Australia

## 

Through advances in therapy and technology, the quality of life, morbidity and mortality outcomes in people with type 1 diabetes continue to improve in countries with well-developed health-care systems, but major disparities in diabetes care exist globally. Ideally, an individual with type 1 diabetes should be managed by a multidisciplinary healthcare team delivering integrated clinical care. Essential to the delivery of optimal diabetes care is education of health care teams, individuals, the community and policy makers. Evidence based clinical care guidelines are an important component of such education. However, while guidelines provide guidance for the practice of evidence based care at the organisational level, evidence based medicine requires the integration of research evidence with clinical expertise and patient values. Since many aspects of health care depend on individual factors such as quality of life, guidelines should be used in the context of the health-care needs and circumstance of each individual with diabetes. Globally, the practicality of implementing evidence based recommendations also depends on the health system structure, availability of resources, economic considerations and socio-cultural factors.

There are a range of guidelines available for the management of young people with type 1 diabetes, including those produced by NICE, ADA, ISPAD and IDF. In Australia, evidence-based guidelines for management of type 1 diabetes across the lifespan were launched in November 2011 [1]. Through the collaborative efforts of the Australasian Paediatric Endocrine Group (http://www.apeg.org.au) and the Australian Diabetes Society (http://www.diabetessociety.com.au), the guidelines address key aspects of diabetes care, based on the best available evidence at the time of writing. The guidelines are structured around clinical questions addressed by systematic reviews and meta-analyses. Notably, evidence for rapidly evolving areas such as use of technologies (pumps, continuous glucose monitoring) is constantly changing. Furthermore, there are aspects of care for which there is little or no evidence, so management is based on best practice or consensus. The applicability of these and other contemporary guidelines to the Asia Pacific Region will be reviewed.
